# Typologies of people’s preexisting political ideology and values would determine their post-pandemic mental health and political behaviors: Evidence from China

**DOI:** 10.3389/fpsyg.2022.1041358

**Published:** 2023-01-06

**Authors:** Shaojie Pan, Xiaoqin Xie, Linghong Xu

**Affiliations:** ^1^The School of Public Administration, Southwestern University of Finance and Economics, Chengdu, China; ^2^The School of Finance, Southwestern University of Finance and Economics, Chengdu, China

**Keywords:** COVID-19, uncertainty, depression, political engagement, latent profile analysis

## Abstract

The pandemic of COVID-19 has caused economic and social crisis across the world. Existing studies have shown that the uncertain social context has profoundly affected people’s life, triggering a variety of social psychological phenomena including the deterioration of mental health and the change of political behavioral patterns. However, little has been known about the differences in people’s pre-pandemic political ideology and their influences on people’s mental health and political behaviors after the pandemic. Using the secondary data from the 2018 and 2020 China Family Panel Studies, we measured nationalism tendencies, state performance ratings, social justice evaluation and life satisfaction of 29,629 adults before the pandemic. Using latent profile analysis (LPA), we examined the typologies of respondents’ political ideology and values. Five types emerged to identify respondents with different political ideology and values: (Class-1) High nationalism tendency, country evaluation, social justice perception, and life satisfaction; (Class-2) Low life satisfaction; (Class-3) Moderate ratings; (Class-4) Low nationalism tendency; and (Class-5) Low country evaluation, low social justice perception. We further explored the predicting roles of those typologies on people’s depressive symptoms and political engagement behaviors after the pandemic. We found that, after the pandemic, although the depressive symptoms of people with low life satisfaction (Class-2) and low country and society ratings (Class-5) eased, they still tended to have more severe depressive symptoms than the Moderate ratings group (Class-3). People with low life satisfaction (Class-2) were also less likely to follow political information than the moderate group (Class-3). Our research revealed how the psychology and behaviors of Chinese people with different political views changed when faced with uncertainty in social context. Further research needs to be carried out to depict how these changes occur.

## 1. Introduction

Breaking out at the end of 2019, the pandemic of COVID-19 has caused huge losses to the world, including 594,367,247 confirmed cases and 6,451,016 deaths by August 23, 2022 (WHO, 2022). COVID-19 also generated uncertainty that rippled around the world. Especially in the economic field, the outbreak of the pandemic has had global effects, including the contraction of production output, adjustment of supply chains, and higher volatility of industries (Caggiano et al., 2020, p. 4; Javorcik, 2020, p. 111; Szczygielski et al., 2022, p. 1). Accordingly, countries including China were likely to experience lower production, consumption and employment, and their economic outlook was full of uncertainties (Altig et al., 2020, p. 9; Iyke, 2020, p. 3).

Individuals’ lives have also been affected by the uncertain economic situation. The most immediate consequence was the loss or change of jobs (Venkatesh, 2020, pp. 2–3; Mueller et al., 2021, p. 1), which made people’s economic outlook become pessimistic. Furthermore, due to the uncertainty stress, people’s health was endangered considering their increased fear of disease and poor sleep quality (Peng et al., 2021, p. 334; Wu D. et al., 2021, p. 1). Additionally, as the policy measures were developed to urge social distancing and home confinement, people’s participation in social relationships were significantly reduced. Coupled with the restrictions on living standards due to the pandemic, people’s life satisfaction dropped to a low point (Ammar et al., 2020, p. 2; Chen and Olsen, 2022, p. 2,281).

The uncertainty of economic status, health status, social relations and quality of life brought about by the epidemic has had a negative impact on people’s mental health and behaviors, such as psychological distress, loneliness, poor resilience, and lower willingness to marry (Rettie and Daniels, 2021, p. 427; Godinić et al., 2020, p. 61; Karataş and Tagay, 2021, p. 1; Parlapani et al., 2020, p. 1; Guetto et al., 2021, p. S674). Actually, existing studies have systematically summarized the psychological impacts of the COVID-19 pandemic. Studies have shown that the COVID-19 pandemic can not only cause mental health problems such as depression, anxiety, frustration, loneliness, post-traumatic stress disorder (PTSD), and obsessive–compulsive disorder (OCD), but also unhealthy or problematic behaviors such as smoking, alcohol dependence, and substance use (Zvolensky et al., 2020, pp. 2–6; Pedrosa et al., 2020, pp. 1–5). In an international study conducted among 9,565 people from 78 countries during the pandemic, 66.9% of respondents reported moderate to high levels of stress, 10% of respondents were psychologically languishing, and 25–50% of respondents showing depressive symptoms such as lack of reinforcement, boredom, and waste time (Gloster et al., 2020, p. 2). Regarding substance use behaviors, taking the United States as an example, researchers surveyed the behavioral health status of 5,412 adults between June 24 and 30, 2020. They found that 13.3% of respondents had started or increased substance use to cope with stress or emotions caused by COVID-19 (Czeisler et al., 2020, p. 1050).

The uncertain social environment created by the COVID-19 pandemic has also changed people’s behavior patterns. For example, people may change their hygiene and social behaviors to avoid being infected with the virus (Barber and Kim, 2021, p. e17; Fong et al., 2020, p. 1), they may also show selfish and non-cooperative behaviors to protect their own interest (Albarracin and Jung, 2021, pp. 12–15; Ling et al., 2020, p. 312). The pandemic also had intense impacts on people’s political behaviors. Due to the fear and anxiety caused by the pandemic, people may exhibit discrimination towards other ethnic groups and hatred to immigrants and foreigners (Meier et al., 2021, p. 403; Reny and Barreto, 2022, p. 209), which further developed into other political trends, such as the rise of support for nationalism and authoritarianism (Diaz and Mountz, 2020, p. 1037; Hartman et al., 2021, p. 1274). The pandemic also exacerbated the political divides among people (Sommer and Rappel-Kroyzer, 2022, p. 769), which had complex effects on people’s political behaviors. Motivated by the anger and anxiety caused by the pandemic, some people intended to have more political engagement to support beneficial policies (Renström and Bäck, 2021, p. 861), while others would even be more inclined to participate in political violence (Bartusevičius et al., 2021, p. 1391). On the contrary, some people would be less willing to vote out of dissatisfaction with the government (Bernardi and Fleischer, 2022, p. 1), and some groups have been politically marginalized because of the plight of the epidemic (Brechenmacher and Hubbard, 2020, pp. 1–4).

If we anticipate changes in people’s mental health and political behaviors caused by the pandemic, it is important to understand what causes the difference in people’s mental health and political behaviors. Existing literature has shown that individuals’ different characteristics, including personality, gender, age, education, income, and type of residence, led to different mental health and political behaviors after the outbreak (Janning et al., 2021, pp. 10–11; O'Connor et al., 2021, p. 326; Alzueta et al., 2021, p. 556; Freitag and Hofstetter, 2021, p. 1; Hampshire et al., 2021, p. 1). Political ideology and values may also play a major role in shaping an individual’s post-pandemic mental health and political behaviors. According to the theories in political psychology, individuals’ ideology and values are important predictors of their political behaviors, which accounts for people’s various political attitudes in contexts (Krosnick et al., 2010, pp. 1307–1310; Jost et al., 2009, p. 183). Accumulating evidence suggests that, once formed, an individual’s political ideology and values can have a multifaceted impact on many aspects of psychological and social functioning, including mental health and political behaviors (Hatemi and McDermott, 2016, p. 331).

Consistent with the political psychology theories, the impacts of political ideology and values on individuals’ mental health and political behaviors has also been demonstrated in uncertain social environment caused by the pandemic. For example, in studies conducted among Americans, people’s political identity (i.e., Democrat or Republican, Support or not for Trump Presidency, left–right ideological positioning) had a strong influence on both their emotional distress and policy preferences during the pandemic (Collins et al., 2021, p. 1; Gadarian et al., 2021, p. 1). Actually, political ideology and values are multidimensional concepts that encompass not only views on economic and society, but also exocentric (i.e., international) and endocentric (i.e., self, social, national aspects) perspectives (Feldman and Huddy, 2014, p. 312; Babula and Muschert, 2021, p. 658). Existing studies showed that political ideology and values, including xenophobia, governmental performance evaluations, perceived fairness, and self-development, demonstrated influences on people’s mental health and political behaviors after the outbreak of COVID-19, which also cover the international, national, social, and individual levels of indicators (Ahuja et al., 2021, p. 46; Belchior and Teixeira, 2021, p. 1; Lu et al., 2021, p. 1925; Islm et al., 2021, p. 1). However, research that examines the profiles of people’s political ideology and values from multiple indicators and explores the relationship between these profiles and people’s mental health and political behavior is very rare. Such research is needed considering the multidimensional nature of political ideology and values and the variety of political ideology and values among people (Carmines and D’Amico 2015, pp. 211–213).

As the first country hit by the pandemic, China has been profoundly affected and changed by the COVID-19 pandemic. The political psychological trends of the Chinese people, including the rapidly rising patriotism, nationalism and xenophobia and high-level trust in government (Wang and Tao, 2021, p. 525; Thomas and Zhang, 2020, p. 1; Xu et al., 2021, p. 51; Wu C. et al., 2021, p. 930), stunned the world and aroused widespread interest in scholars from all over the world. There have been also studies exploring the impacts of political ideology and values on mental health and political behaviors among Chinese people. For example, a study conducted among 745 American and Chinese college students found that trust in government positively predicted mental health outcomes only among Chinese participants (Wang et al., 2022, p. 1). Another study using an online survey believed that nationalism contributed to participants’ behaviors of following the advocation of the government (Jia and Luo, 2021, pp. 6–7). However, those studies were predominately cross-sectional studies to explore the concurrent relationship between political ideology and values and mental health or political behaviors during the pandemic, failing to reveal people’s pre-existing political ideology profiles before the pandemic and their influences on mental health and political behaviors after the pandemic. Nor can those cross-sectional studies effectively establish causal relationships.

Building on existing literature, this study anticipated multiple types of political ideology and values among Chinese people. Specifically, based on longitudinal data, we explored the profiles of Chinese people’s political ideology and values before the outbreak, as indicated by measurements from the international, national, social, and individual aspects. To filling the research gaps, we further sought to build and test a relationship between people’s pre-pandemic political ideology and values and post-pandemic mental health and political behaviors. We also incorporated sociodemographic characteristics into the analysis to better understand the mechanism of social psychological changes during the pandemic.

## 2. Materials and methods

This research bases analysis on data from the 2018 and 2020 China Family Panel Studies (CFPS). CFPS was conducted with a baseline survey in 2010 and a full-sample follow-up survey every 2 years thereafter. Using the probability proportional to size (PPS) sampling method, CFPS administered a base-line sample of 14,960 households, 42,590 individuals in 25 provincial-level administrative regions of China, representing 95% of China’s population (Xie et al., 2017, p. 1). In order to explore the psychological and behavioral changes of people with different political positions before and after the pandemic, this article reports analysis of data from the 4th (2018) and 5th (2020) waves of this longitudinal study. Specifically, the 2018 survey was conducted between June 2018 and March 2019, and the 2020 survey started in July 2020 and ended in December 2020 (Wu et al., 2020, p. 2).

The research sample consisted of 29,629 adults. In fact, of the 42,590 participants included in the 2010 baseline survey, 33,973 had reached adulthood in 2018. In this study, however, we only included participants who were measured with political ideology and values in 2018 and mental health status and political engagement behaviors in 2020, resulting in a sample of 29,629. The sociodemographic characteristics of the research sample in 2018 are as follows. The mean age was 47.41 years (SD = 16.42) and slightly over half (50.45%) of the sample were female. Nearly three-quarters (73.73%) of the sample had rural household registration while the rest (26.27%) had urban household registration. Most (80.55%) of the sample were either married or living with a partner, and 19.45% were single, divorced, or widowed. The respondents also reported their highest level of education, with approximately one tenth (12.24%) of them maintained a college degree or more.

This study used the open-access data from CFPS 2018 and 2020 after the application to the Institute of Social Science Survey of Peking University had been approved. The Ethical approvals for CFPS 2018 and 2020 were provided by the Institutional Review Board of Peking University.

### 2.1. Measurements

#### 2.1.1. Indicators for latent profiles

Four indicator variables measured in 2018 were used to explore the latent profiles of Chinese adults regarding political ideology and values. These four variables demonstrated respondents’ nationalism tendencies, evaluations of the country, perceptions of social justice, and life satisfaction, respectively. (1) Nationalism tendency. A single item, “How much do you trust Americans?,” was used as the measurement. Respondents rated on a 11-point Likert scale from 0 (Distrustful) to 10 (Very trustworthy) to indicate their attitudes. Because there have been many economic and political conflicts between China and the United States in recent years, and studies have shown that events such as the trade war have caused Chinese people’s trust in the United States to decline and nationalist tendencies to rise (Sha, 2021, p. 1), we adopted the anti-Americanism sentiment as the measurement for nationalism tendencies. This item was reversely coded with higher scores indicating stronger nationalism tendencies. (2) Evaluation of the country. The average score of eight items served as the measurement. The eight items asked respondents to rate the severity of the eight problems in China, about the environment, the inequality between the rich and the poor, employment, education, medical service, housing, social security, and government corruption. Each item was measured on a 11-point scale, from 0 (Not severe) to 10 (extremely severe). We reverse-coded these items so that a higher score indicated a higher assessment of the country by respondents. This measurement demonstrated satisfactory reliability (Cronbach’s alpha = 0.86). (3) Perception of social justice. The composite score of two items was administered to respondents, including “Hard work will be rewarded in today’s society” and “Intelligence and wisdom will be rewarded in today’s society.” Responses to each item ranged from 1 (Strongly disagree) to 5 (Strongly agree) with the composite score ranging between 2 and 10. Higher scores suggested respondents’ better perceptions of social justice. (4) Life satisfaction. A single item, “Are you satisfied with your life?,” was used to assess life satisfaction. Respondents’ answers ranged from 0 (Least satisfactory) to 10 (Most satisfactory).

#### 2.1.2. Covariates

Respondents’ socio-demographic information in 2018 was controlled, including age, gender (man or woman), household registration (rural or urban), highest education level (high school or less, college or more), and marital status (married/living with a partner or single/divorced/widowed). The income of respondents was not included in the analysis due to the high data missing proportion.

#### 2.1.3. Dependent variables

Two indicators were utilized to measure respondents’ depressive symptoms and political engagement behaviors in both 2018 and 2020. A simplified version of the Center for Epidemiologic Studies Depression Scale (CES-D) was used to assess respondents’ depressive symptoms (Radloff, 1977, p. 385). The simplified CES-D scale consisted of eight items, measuring depressive symptoms such as “feel sad” and “cannot sleep well.” Respondents were asked to rate on a 4-point Likert scale, from 1 (Never) to 4 (Most of the time). The range of cumulative values of the eight items was 8–32, and higher values indicated severer depressive symptoms. This measurement demonstrated its high reliability in both 2018 (Cronbach’s alpha = 0.77) and 2020 (Cronbach’s alpha = 0.77) waves.

A composite score of two questions was used to measure the frequency of respondents to obtain political news. The two questions asked respondents how many days in the past week they obtained political news through television and internet. Respondents’ answer to each question ranged between 0 and 7 (days) and the composite score ranged from 0 to 14. Higher values suggested that respondents were keener on getting political news.

### 2.2. Analysis

Data analysis was performed on Stata 16.0. First, latent profile analysis (LPA) was conducted to explore the typologies of political ideology and values among the respondents before the COVID-19 pandemic. Based on four indicators, a two-profile model was first constructed, and one additional profile was added to each subsequent model. We estimated models iteratively and examined the fit statistics of each model to identify the model with optimal numbers of profiles. The fit statistics we used in the analysis included the Akaike Information Criterion (AIC) and the Bayesian Information Criterion (BIC), with the lowest value indicating the best goodness of fit among all models (Vrieze, 2012, p. 228). After the best-fit latent profile models had been determined, each participant was assigned membership to a latent profile if their probability of belonging to this profile was highest. Then we also presented the prevalence of each profile among the participants.

Second, upon the selection of the optimal latent profile model, multinomial regression was performed to depict the social demographic characteristics of each profile. Specifically, covariates including age, gender, household registration, education level, and marital status were used to predict latent profile membership. The multinomial regression model can be written as Equation (1). To illustrate, *p_i_* = the probability of falling into Class-1, Class-2, Class-4, or Class-5, *p_c3_* = the probability of belonging to the reference group (Class-3), and *X_1_*–*X_5_* represent the covariates.


(1) $$\begin{array}{l} \mathrm{mLogit}\left(p_{i}\right)=\ln\left(p_{i}/p_{c3}\right)=\alpha_{1}+\beta_{11}X_{1}+\beta_{12}X_{2}+\\ \beta_{13}X_{3}+\\ \beta_{14}X_{4}+\beta_{15}X_{5}+\varepsilon \end{array}$$

Finally, linear regressions were performed to explore the relationship between respondents’ pre-pandemic typologies of political ideology and values and post-pandemic depressive symptoms and political engagement behaviors. The aforementioned sociodemographic variables and respondents’ pre-pandemic depressive symptoms and political engagement behaviors were also controlled in the regression models. The linear regression models for depressive symptoms and political engagement behaviors are also written in equation form, as shown in Equations 2, 3, respectively. Specifically, X_C1_–X_C5_ are values of the dummy variables for latent profiles, *X_1_*–*X_5_* represent the values of covariates, and *X_DS2018_* and *X_PE2018_* indicates the values of depressive symptoms and political engagement behaviors in 2018.


(2) $$\begin{array}{l} Y_{DS}=\alpha_{2}+\beta_{21}X_{C1}+\beta_{22}X_{C2}+\beta_{23}X_{C4}+\beta_{24}X_{C5}+\\ \beta_{25}X_{1}+\beta_{26}X_{2}+\beta_{27}X_{3}+\beta_{28}X_{4}+\\ \beta_{29}X_{5}+\beta_{210}X_{DS2018}+\beta_{211}X_{PE2018}+\varepsilon \end{array}$$

(3) $$\begin{array}{l} Y_{PE}=\alpha_{3}+\beta_{31}X_{C1}+\beta_{32}X_{C2}+\beta_{33}X_{C4}+\beta_{34}X_{C5}+\\ \beta_{35}X_{1}+\beta_{36}X_{2}+\beta_{37}X_{3}+\beta_{38}X_{4}+\\ \beta_{39}X_{5}+\beta_{310}X_{DS2018}+\beta_{311}X_{PE2018}+\varepsilon \end{array}$$

By using *t*-test and chi-square analysis, we also compared the mean values of participants’ depressive symptoms and political engagement behaviors before and after the pandemic to present the social psychological changes of different latent groups.

## 3. Results

Table 1 presents the descriptive characteristics of the research sample, which are also introduced in the Methods section. Table 2 shows the fit statistics from the model with one class to the model with six classes. The two indicators we use to select the optimal number of classes, the Akaike Information Criterion (AIC) and the Bayesian Information Criterion (BIC), represent a better fit when exhibiting a lower value (Tein et al., 2013, p. 640). As seen from Table 2, the values of AIC and BIC both decrease continuously from the 1-class model to the 5-class model, and then increase again to the 6-class model, making the 5-class model showing the best fit results.

**Table 1:** Social demographic characteristics of the sample.

		*N*	%
Gender	Women	14,949	50.45
	Men	14,680	49.55
Age	18–29	5,316	17.94
	30–39	5,025	16.96
	40–49	5,404	18.24
	50–59	5,963	20.13
	60–69	5,097	17.20
	70–99	2,824	9.53
Household Registration	Rural	21,818	73.73
	Urban	7,772	26.27
Education	High School or Less	26,003	87.76
	College or More	3,626	12.24
Marital Status	Married/Living with a partner	23,867	80.55
	Single/Divorced/Widowed	5,762	19.45

**Table 2:** Comparison of latent profile analysis fit statistics.

Model	df	LL	AIC	BIC
1 Class	8	−241898.2	483812.3	483878.7
2 Classes	13	−240272.1	480570.1	480678.0
3 Classes	18	−237034.1	474104.2	474253.5
4 Classes	23	−236320.7	472687.5	472878.3
5 Classes	28	−236141.3	**472338.5**	**472570.8**
6 Classes	33	−236141.3	472348.5	472622.3

df, degree of freedom; LL, log likelihood; AIC, Akaike Information Criterion; BIC, Bayesian Information Criterion.The bold values indicated the best-fitted model because the least AIC or BIC values indicate the optimal number of classes.

**Figure 1:**
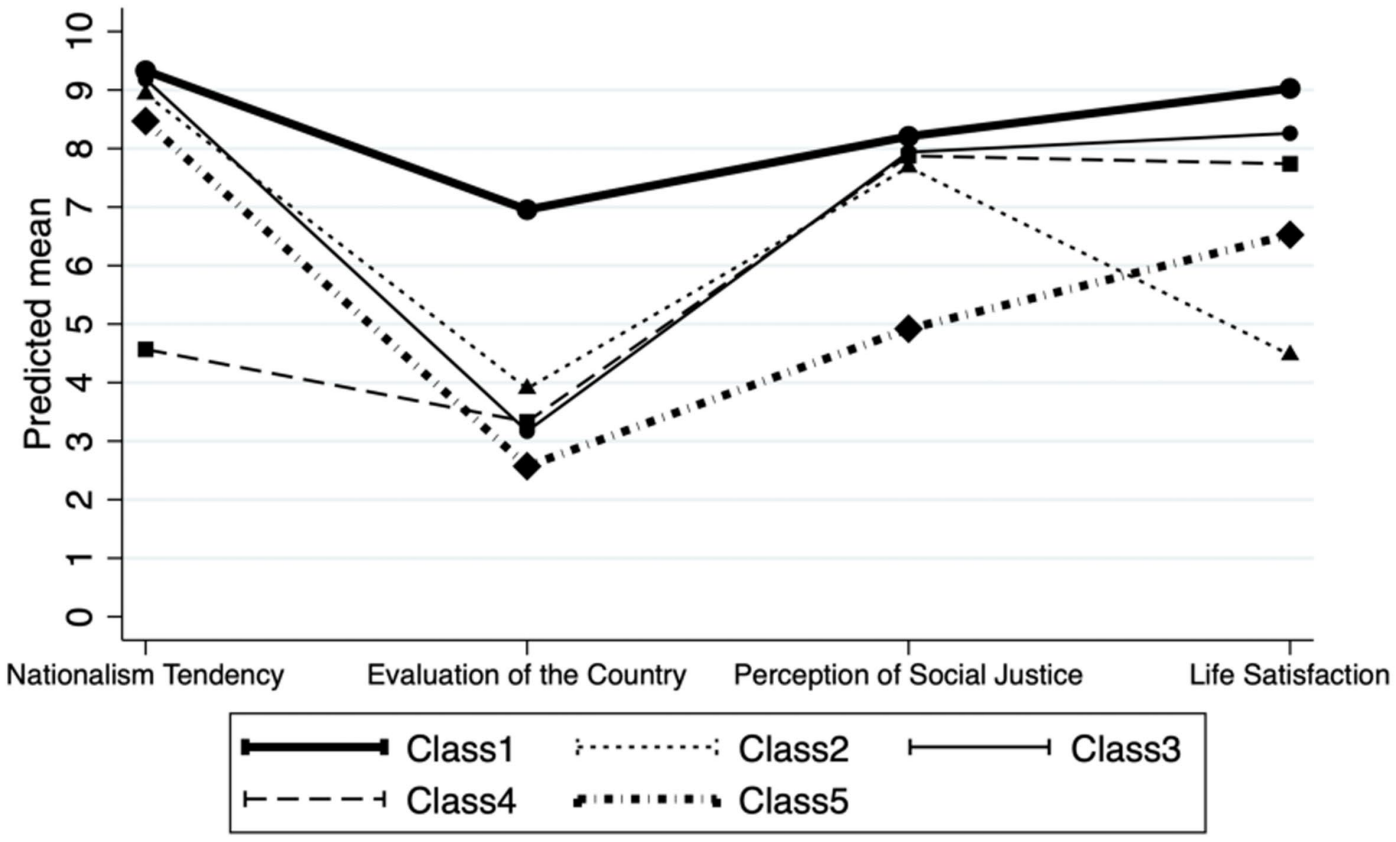
Political ideology and value by class.

As presented in Figure 1, the 5-class solution revealed distinct profiles of political ideology and values among respondents. Specifically, Class-1 represents people who rated the highest scores on all indicators. Members of Class-2, Class-3, and Classs-4 showed similar scores on both evaluation of the country and perception of social justice, but they differed in that Class-2 had the lowest score for life satisfaction, while Class-4 had the lowest score on nationalism tendency. At the same time, Class-3 appeared to give moderate rating on all indicators. Members of Class-5 are distinguished in that they had the lowest scores on both evaluation of the country and perception of social justice, and the second-lowest scores on both nationalism tendency and life satisfaction.

Class-1 (high nationalism tendency, country evaluation, social justice perception, and life satisfaction) is the smallest group consisting of only 2.4% of the sample. Members in this group were characterized by demonstrating the strongest nationalist sentiments, speaking highly of the country and society, and also being very satisfied with their own lives. In particular, they rated the country’s performance almost twice as high as the people in other classes.

Class-2 (low life satisfaction) constitutes 12.8% of the sample. For members in this group, they were similar to other classes regarding nationalism tendency, country evaluation, and social justice perception, but distinguished themselves from other classes because of the obviously lowest life satisfaction.

Class-3 (moderate nationalism tendency, country evaluation, social justice perception, and life satisfaction) become the largest category accounting for 41.7% of the sample. Members in this group appeared to report average scores across the measurements for political ideology and values. This class has also become the reference group in our multiple regression models.

Slightly over one third (36.4%) of the respondents fell into Class-4 (low nationalism tendency). This category represents people who held lower nationalist sentiments and maintained a more friendly attitude towards foreigners. Like most others, members of this group rated the country lower, social justice higher, and had higher-than-average personal life satisfaction.

Approximately 6.8% of the sample belonged to Class-5 (low country evaluation, low social justice perception). Members of this class seemed to have a relatively pessimistic attitude towards everything. Not only were they the least likely to recognize the state’s performance, but their perception of social justice was far lower than that of other groups. Moreover, their life satisfaction was also very low, only higher than people in Class-2.

Multinomial logistic regression was performed to test if the members of the latent classes differed by age, gender, household registration, education, and marital status. As displayed in Table 3, older people, those with rural household registration, those who had no college education, and those who were single, divorced or widowed have a significantly greater chance of belonging to Class-1, as compared to Class-3. For Class-2, it’s members were more likely to be female, people with rural household registration, people without college education, and people who were single, divorced or widowed, as compared to Class-3. Members in Class-4 tended to be younger people, those with urban household registration, those with college education, and those who were single, divorced or widowed when compared to their counterparts in Class-3. Lastly, younger people, people with urban household registration, and people who attended college had a significantly higher probability to enter Class-5, as compared to Class-3.

**Table 3:** Multinomial logistic regression predicting class membership by social demographics.

Social demographic variables	Class 1		Class 2		Class 4		Class 5	
	*b*(SE)	RRR	*b*(SE)	RRR	*b*(SE)	RRR	*b*(SE)	RRR
Age	0.04(0.00)	1.04^***^	0.01(0.00)	1.00^***^	−0.01(0.00)	0.99^***^	−0.00(0.00)	1.00^*^
Gender (Male)	0.19(0.10)	1.21	−0.09(0.04)	0.91^*^	−0.02(0.03)	0.98	−0.02(0.05)	0.98
Household registration (Urban)	−0.77(0.15)	0.46^***^	−0.56(0.05)	0.57^***^	0.09(0.03)	1.09^**^	0.79(0.06)	2.21^***^
Education (College or more)	−1.19(0.46)	0.30^*^	−0.64(0.09)	0.53^***^	0.60(0.04)	1.81^***^	0.25(0.08)	1.28^**^
Marital status (Married or living with a partner)	−0.30(0.14)	0.74^*^	−0.59(0.05)	0.55^***^	−0.57(0.03)	0.56^***^	−0.13(0.07)	0.88
	Model Fit							
	χ ^2^	*df*	*p*					
Likelihood ratio test	2133.17	20	<0.001					

The reference group was Class 3. RRR, Relative Risk Ratio. ^*^p < 0.05, ^**^p < 0.01, ^***^p < 0.001.

Having identified different profiles of people’s pre-pandemic views on politics and society, we sought to discover how they relate to people’s different mental health and political behaviors after the pandemic. Even after controlling for pre-pandemic mental health, members of Class-2 and Class-5 still had significantly severer symptoms of depression after the pandemic than the reference group (Class-3). Particularly, people in Class-2 seemed to have even worse post-pandemic depressive symptoms than those in Class-5. As compared to Class-3, members in Class-2 exhibited a significantly lower frequency of obtaining political news than members in Class-3, demonstrating the lowest willingness to pay attention to politics among all categories. The finding remained unchanged after incorporating the frequency of people’s obtaining news before the pandemic into the model (Table 4).

**Table 4:** Results of linear regressions predicting mental health and political engagement behaviors.

	Depressive symptoms (CES-D)		Frequency of obtaining political news	
	*b*(SE)	Beta	*b*(SE)	Beta
Class 1	0.46(0.26)	0.01	−0.51(0.27)	−0.01
Class 2	0.94(0.09)	0.08^***^	−0.48(0.09)	−0.04^***^
Class 4	−0.08(0.06)	−0.01	0.06(0.06)	0.01
Class 5	0.59(0.12)	0.03^***^	0.00(0.12)	0.00
Age	0.00(0.00)	0.01	−0.01(0.00)	−0.04^***^
Gender (Male)	−0.35(0.05)	−0.04^***^	0.80(0.06)	0.09^***^
Household registration (Urban)	−0.46(0.07)	−0.05^***^	1.22(0.07)	0.12^***^
Education (College or more)	−0.11(0.09)	−0.01	0.79(0.09)	0.06^***^
Marriage status (Married or living with a partner)	−0.17(0.07)	−0.02^*^	−0.00(0.08)	−0.00
Depressive symptoms in 2018	0.48(0.01)	0.45^***^	–	–
Frequency of obtaining political news in 2018	–	–	0.43(0.01)	0.42^***^
Adjusted *R*^2^	0.25		0.27	

The reference group was Class 3. ^*^p < 0.05, ^**^p < 0.01, ^***^p < 0.001.

Table 5 also presents the different trends of mental health and political behavior changes in different latent groups. Overall, depression in all participants increased after the pandemic. Specifically, Class-1 and Class-3 had significantly increased depressive symptoms while Class-2 and Class-5 had decreased symptoms, with Class-4 almost remaining unchanged. We also witnessed an increase of frequency of obtaining political news among all participants after the pandemic. Specifically, except for Class-1, which basically did not change, the participants in other categories all showed a trend of significant increase.

**Table 5:** Post-pandemic changes of mental health and political engagement behaviors by latent profiles.

	Depressive symptoms (CES-D) (Mean)		Frequency of obtaining political news (Mean)	
	Pre-pandemic	Post-pandemic	Pre-pandemic	Post-pandemic
Class 1	12.26	13.41^***^	4.16	4.18
Class 2	15.87	15.59^**^	3.40	4.00^***^
Class 3	12.88	13.16^***^	4.59	5.17^***^
Class 4	13.21	13.22	5.01	5.59^***^
Class 5	15.23	14.79^**^	5.04	5.65^***^
All	13.49	13.58^**^	4.61	5.19^***^

^*^p < 0.05, ^**^p < 0.01, ^***^p < 0.001.

## 4. Discussion

Based on longitudinal data, this study aims to depict the different preexisting patterns of Chinese people’s political ideology and values and the subsequent psychological and behavior consequences in the uncertain social context caused by the COVID-19 pandemic. Firstly, indicated by nationalism tendency, evaluation of the country, perception of social justice, and life satisfaction, 5 latent profiles emerged to describe Chinese people’s political ideology and values before the outbreak of COVID-19, including the high nationalism tendency, country evaluation, social justice perception, and life satisfaction group (Class-1), low life satisfaction group (Class-2), moderate ratings group (Class-3), low nationalism tendency group (Class-4), and low country evaluation and social justice perception group (Class-5).

Our study found that most groups scored higher on nationalist tendencies, concentrating around 9 points (Full score: 10 points). This is in concert with prior studies that mentioned the high nationalism sentiment in China in recent years (Wang and Tao, 2021, p. 525; Weiss, 2019, p. 1). Because trust in Americans was used as the measurement of nationalist tendency, our research found that Chinese anti-American nationalist sentiment was already at a high level before the pandemic. It could be related to economic and political conflicts between the two countries in 2018, such as Trade War (Boylan et al., 2021, p. 23). At the same time, however, groups with high nationalism tendency, country evaluation, social justice perception, and life satisfaction only constituted a very small percentage of all participants (Class-1, 2.4%). This is an interesting finding considering the disproportionate attention given to the highly nationalistic group in China, such as the “Little Pinks” (Wei and Chen, 2022, p. 25). Our research shows that the group with very high nationalist sentiments were only a tiny fraction of the overall population, while a large percentage of Chinese still maintained low nationalist tendencies (Class-4, 36.4%). Moreover, contrary to popular belief, we found that people with high nationalism tendency tended to be older while people holding low nationalism sentiment were younger when compared to the moderate ratings group (Class-3, 41.7%). This further shatter the stereotype that the fervent proponents of nationalism in China are young people or “Little Pinks” and corresponded to the conclusion of Li’s (2015) article. However, further research still needs to explore whether the above-mentioned nationalism tendencies in Chinese people have changed after the pandemic.

We also found that most of the participants (Class-2, Class-3, Class-4, Class-5) rated the performance of the country on a low level below 4 points (Full score: 10 points). This suggests that even high level of nationalism do not naturally lead to high level of satisfaction and trust in the country or government. For example, through analyzing tweets from 146 Chinese opinion leaders on Weibo, Zhang et al. (2018) argued that nationalist may also incorporate liberal values to challenge the government rather than unconditionally support for the regime. But on the other hand, there are also studies showing that Chinese citizens were very satisfied with the government during the epidemic, with an average rating of 3.8 (Full score: 5 points; Wu C. et al., 2021, p. 8). In this sense, Chinese people’ evaluation with the country and the government may have increased significantly during the epidemic.

Two categories emerged to describe people with more negative political and social perspectives. 12.8% of the participants (Class-2) significantly held much lower life satisfaction than other groups. Moreover, compared with the largest group with moderate ratings (Class-3), people in Class-2 were more likely to be female, people with rural household registration, people without college education, and people who were single, divorced or widowed. Prior studies had mixed results about the impacts of gender, household registration status, education, and marital status on life satisfaction and similar constructs (Liang and Shen, 2016, p. 1; Zhang, 2010, p. 19; Zhang et al., 2022, pp. 1–2). But our research showed that female, rural, low-educated, unmarried or divorced people may still constitute the most disadvantaged group in China. Our research also partly supported the conclusion of Liu et al. (2021) that Chinese with stronger authoritarian ideology and national identity tend to have higher life satisfaction. Compared with people rating highest across all indicators (Class-1), members in Class-2 demonstrated significantly lower ratings on country evaluation and life satisfaction. Their lower national satisfaction and lower life satisfaction may be closely related.

Class-5 accounted for 6.8% of the participants who had the second-lowest nationalism tendency and life satisfaction and lowest country evaluation and social justice perception. This category represents a particular Chinese youth group, “Fen Qing,” or in English, the “angry youths.” They represent young Chinese people who vigorously expressed and even acted on political and social issues. In the existing literature, researchers divided “angry youths” into three types: nationalism, China-critical, and resentment-venting, or two types, nationalism and liberalism, according to their positions. The liberal or China-Critical angry youths may tend to speak highly of America or democracy and criticize the Chinese government while the nationalist angry youths may be anti-American and express hatred to western countries (Yang and Zheng, 2012, p. 637; Osnos et al., 2009). However, our research found that in 2018, angry youths in China also maintained a high level of nationalist sentiment despite their critical perspective. Their nationalist tendency remained at around 8.5 points (Full score: 10 points) while their ratings of country performance, social justice and personal life were all low. We also found that Class-5 tended to include younger, urban, and highly educated people when compared to Class-3. This is also in line with Woods and Dickson (2017), as they claimed that China’s angry youths are mostly well-educated young people from middle-class families.

Our research demonstrated that Class-2 and Class-5, representing people with lowest satisfaction and people holding a critical perspective on the country and society, showed more severe depressive symptoms after the pandemic. But we also found that Class-2 and Class-5 were the only two groups whose depressive symptoms were relieved after the pandemic. In other words, the mental health of the members of Class 2 and Class 5 had eased through the pandemic, but they still had more depressive symptoms than those of other groups. First, considering participants’ worsening mental health overall, it was an interesting finding that Class-2 and Class-5 showed reduced depressive symptoms post-pandemic. The measurement of participants’ post-pandemic depressive symptoms was conducted in the second half of 2020. At that time, China’s pandemic prevention and control strategy had achieved a great success (He et al., 2020, p. 242) and people’s lives were gradually returning to normal after the first wave of the pandemic. For the members of Class-2 and Class-5, perhaps it was the feeling of the effectiveness of the pandemic prevention policy that made them more positive (Chu et al., 2021, p. 1). Moreover, the patriotic feelings generated by trust in the government may also have a protective effect on their mental health (Wang et al., 2021, p. 1), preventing them from developing more depression. But people belonging to Class-2 and Class-5 still tended to have worse mental health condition when compared to other categories. Class-2 members maintained the highest levels of depression both before and after the pandemic. Taking into account their disadvantaged status regarding gender, household registration, and marital status, their poorest mental status may be due to the lack of significant improvement in their socioeconomic status and living conditions. Members of Class 5 maintained the second-highest depression scores before and after the pandemic. For them, perhaps the effective performance of the government during the pandemic had generated some positive emotions, but their critical perspective and the resulting negative emotions had not changed significantly.

Class-2 membership was significantly associated with lower frequency of obtaining political news after the pandemic. It seems that although the pandemic tended to increase the frequency of following political news of all people, those with low life satisfaction still had significantly lower political attention than other groups after the pandemic. It is not a novel finding that people with lower life satisfaction tend to be less politically engaged, considering this conclusion drawn in both Chinese and foreign studies (Appleton and Song, 2008, p. 2325; Flavin and Keane, 2012, p. 63). But data also revealed that Class-2 was mainly composed of women, rural, less educated and single, divorced, or widowed people. It might be the case that those more disadvantaged people were politically marginalized during the pandemic because of increasing economic precarity, reinforcing male political dominance, inequities in digital access, and decreased public visibility (Brechenmacher and Hubbard, 2020, pp. 1–4).

### 4.1. Limitation

The results of our studies should be considered in view of the following limitations. First, we only used limited number of indicators to measure participants’ political ideology and values, which limits a more comprehensive presentation of the participant’s perspectives. For example, our descriptions of participants’ political perspectives can be enriched by incorporating measures of authoritarian support and xenophobia. This is also reflected in the measurement of nationalism tendency. The complex and rich connotation of the concept “nationalism” cannot be captured in the mere use of anti-Americanism sentiment as a measure of nationalism tendency. Second, by using a panel data, we preliminarily examined the relationship between the types of participants’ pre-pandemic political views and their post-pandemic mental health and political engagement behaviors. However, panel conditioning biases may occur when the participants’ responses did not accurately reflect their attitudes or behaviors. For example, the participants’ political attitudes and behaviors may appear to change across different waves only because they were more adapted to and familiar with the survey (Warren and Halpern-Manners, 2012, pp. 499–504). In this sense, future research is needed to investigate panel conditioning effects in the CFPS data to secure those research findings based on this dataset. Last, we preliminarily examined the relationship between the types of participants’ pre-pandemic political views and their post-pandemic depressive symptoms and political engagement behaviors. However, it is possible for people’s political views to change post-pandemic. In this sense, future research is needed to explore the latent types of Chinese peoples’ political views after the pandemic.

### 4.2. Conclusion

The COVID-19 pandemic has created great uncertainty in both social environment and our lives. Our research identified the types of political ideology and values among Chinese people before the pandemic and revealed the predicting effects of those types on post-pandemic mental health and political engagement behaviors. Overall, the COVID-19 pandemic has caused a deterioration in people’s mental health and an increase in political attention. However, people with low life satisfaction and people with critical political ideology and values distinguished themselves from the rest of the population because they not only had worse mental health, but also maintained lower levels of political attention than other groups, respectively. Future research would benefit from understanding the mechanism of psychosocial changes in these two groups during the pandemic.

## Data availability statement

Publicly available datasets were analyzed in this study. This data can be found at: https://opendata.pku.edu.cn/dataverse/CFPS?language=en.

## Author contributions

SP was responsible for literature review, data analysis, and results presentation. XX collaboratively developed the research idea with SP and contributed to the discussion part. LX was responsible for improving the writing and ensuring that the article is formatted correctly. All authors contributed to the article and approved the submitted version.

## Conflict of interest

The authors declare that the research was conducted in the absence of any commercial or financial relationships that could be construed as a potential conflict of interest.

## Publisher’s note

All claims expressed in this article are solely those of the authors and do not necessarily represent those of their affiliated organizations, or those of the publisher, the editors and the reviewers. Any product that may be evaluated in this article, or claim that may be made by its manufacturer, is not guaranteed or endorsed by the publisher.
